# Similar Squamous Cell Carcinoma Epithelium microRNA Expression in Never Smokers and Ever Smokers

**DOI:** 10.1371/journal.pone.0141695

**Published:** 2015-11-06

**Authors:** Antonia Kolokythas, Yalu Zhou, Joel L. Schwartz, Guy R. Adami

**Affiliations:** 1 Department of Oral and Maxillofacial Surgery, College of Dentistry, University of Illinois at Chicago, 801 South Paulina Street, Chicago, Illinois, 60610, United States of America; 2 Arphion Ltd, 2242 W. Harrison Street, Chicago, Illinois, 60612, United States of America; 3 Department of Oral Medicine and Oral Diagnostics, Center for Molecular Biology of Oral Diseases, University of Illinois at Chicago, 801 South Paulina Street, Chicago, Illinois, 60610, United States of America; School of Medicine, Fu Jen Catholic University, TAIWAN

## Abstract

The incidence of oral tumors in patients who never used mutagenic agents such as tobacco is increasing. In an effort to better understand these tumors we studied microRNA (miRNA) expression in tumor epithelium of never tobacco users, tumor epithelium of ever tobacco users, and nonpathological control oral epithelium. A comparison of levels among 372 miRNAs in 12 never tobacco users with oral squamous cell carcinoma (OSCC) versus 10 healthy controls was made using the reverse transcription quantitative polymerase chain reaction. A similar analysis was done with 8 ever tobacco users with OSCC. These comparisons revealed miR-10b-5p, miR-196a-5p, and miR-31-5p as enriched in the tumor epithelium in OSCC of both never and ever tobacco users. Examination of The Cancer Genome Atlas (TCGA) project miRNA data on 305 OSCCs and 30 controls revealed 100% of those miRNAs enriched in never smoker OSCCs in this patient group were also enriched in ever smoker OSCCs. Nonsupervised clustering of TCGA OSCCs was suggestive of two or four subgroups of tumors based on miRNA levels with limited evidence for differences in tobacco exposure among the groups. Results from both patient groups together stress the importance of miR196a-5p in OSCC malignancy in both never and ever smokers, and emphasize the overall similarity of miRNA expression in OSCCs in these two risk groups. It implies that there may be great similarity in etiology of OSCC in never and ever smokers and that classifying OSCC based on tobacco exposure may not be helpful in the clinic.

## Introduction

**MicroRNAs (m**iRNAs) in mature form are noncoding RNAs, 19 to 25 nucleotides in length, with the ability to inhibit the translation and shorten the half-life of mRNAs [1]. MiRNAs can directly regulate multiple mRNAs, which encode the proteins that control important cellular processes. Many of these regulated pathways, including apoptosis, cell proliferation, and cell migration, can also contribute to cancer [2–4]. There are over 2000 known miRNAs, a subset of which have been shown to show changes in levels that correlate with various cancers [5, 6] (http://mirbase.org/). Global expression analysis of these miRNAs in different cancers has identified miRNAs that function as oncomirs, like miR-21-5p, and are consistently upregulated in some cancer types, while other miRNAs are reduced in certain tumor types and appear to be tumor suppressors [7]. Various tumor types have been characterized to show a signature of miRNA levels associated with these tumors and their progression, which may aid in diagnosis and prognosis [5, 6]. The small size and regulatory function of miRNAs have also made them the focus of research using them or similar molecules to change tumor cell properties and thus treat cancer [8–10].

Much effort has gone into describing a set of miRNAs that show consistent changes in levels, first with head and neck squamous cell carcinomas (HNSCCs) [11–13] and more recently with subsets of these cancers such as oral squamous cell carcinoma (OSCC) [14]. The results of these analyses have shown some consistencies, such as fairly universal upregulation of miR-21-5p, and somewhat lower consensus on other potential oncomirs, probably due to the variable amount of mixed epithelium/stroma in samples and diversity of etiology of the tumor subtypes. For example, oral pharyngeal cancer, unlike OSCC even in nonsmokers, is often associated etiologically with transforming HPV, specifically HPV16 [15–17]. Recent work has brought to light two distinct etiologies of OSCC, those associated with the main risk factor known, tobacco usage, and those not [18–20]. Like small-cell lung cancer in never smokers, OSCCs in never tobacco users seem to be distinct [14]. Hereafter, in this report, we will abbreviate this group who do not use tobacco, or other mutagenic products such as betel nut, to “never smokers”. OSCC in never smokers, which is on the increase in the United States, seems to strike on average both older and younger patients than those associated with tobacco usage. It also tends to present in earlier stage, and occurs most frequently in the tongue and gingiva not the floor-of-mouth where tobacco-associated OSCCs occur. Molecularly, OSCCs in never smokers show lower rates of p53 gene mutations, and there is some evidence of differences in gene expression [19–22]. Like tobacco-related OSCCs they are rarely associated with transforming HPV or enrichment of the p16 tumor suppressor [23–26]. Fewer than 10% of oral cancers are HPV gene expression positive even in patients with no history of tobacco use [24, 26]. Overall little is known about the etiology or the changes in mRNA or miRNA associated with this subtype of OSCC [24]

We quantified levels of 372 miRNAs in 12 OSCC epithelial samples from never smokers versus 10 samples from a control group of subjects with apparently normal oral mucosa. We also tested levels of these miRNAs in a test group of OSCCs associated with tobacco usage. Next, we did similar comparisons using the miRNA expression data of the 344 control and OSCC samples from The Cancer Genome Atlas (TGCA) HNSCC cohort. Tumor samples of this study were dissected surgically and contain some stromal tissue. Together we used these datasets to compare miRNA expression in never and ever smokers with the goal of starting to gain insight on etiology of OSCC in these two different OSCC risk groups.

## Materials and Methods

### Clinical sampling

Two brush cytology samples each were collected from 12 subjects who never used tobacco, or other mutagenic agents like betel nut, and who presented with oral lesions that were biopsy-proven OSCC. These patients were seen in the Oral and Maxillofacial Surgery Clinic in the University of Illinois Medical Center. Samples from normal controls were from 10 never tobacco users from oral sites that were normal on clinical examination by the oral surgeon. The second group of brush cytology samples was taken from 18 current or former tobacco users at lesion sites of either OSCC or nonmalignant disease with intact mucosa. Benign samples included mucosal lesions such as leukoplakia, all without dysplasia. All diagnoses were verified by histopathologic examination of surgically obtained tumor tissue for OSCCs and scalpel biopsy material for non-malignant lesions. All subjects in all groups provided written consent to participate in accordance with guidelines of the Office for the Protection of Research Subjects of the University of Illinois at Chicago, the local Institutional Review Board that formally approved of this research.

### Brush cytology

Brush cytology was performed on patients as they presented in the clinic just prior to biopsy as described earlier, taking care to sample areas with intact epithelium [27]. Samples were immediately placed in Trizol (Life Technologies, Carlsbad, CA, USA), mixed, and frozen. We used a cervical cytology brush with RNA purification as described in Schwartz et al. [27].

### RNA

Recent publications have stressed the problems with usage of Trizol to isolate miRNA with ethanol or isopropanol precipitations when RNA levels show a wide range [28]. While cells were stored in Trizol, we used a methodology similar to that recommended by Kim et al. and immediately following phase separation all samples were subjected to silicate-based binding purification to prevent selective miRNA loss (S1 Fig). We used RNeasy chromatography (Qiagen, Germantown, MD, USA) to remove mRNA followed by ethanol addition and RNeasy MinElute chromatography (Qiagen) to bind then elute small RNAs, including mature miRNA. There was a 6-fold range in sample RNA levels based on RT-PCR with similar average levels in the malignant and nonmalignant groups.

### Quantitative RT-PCR

10 ng RNA was reverse transcribed in 5 ul reactions using the miRCURY LNA Universal RT microRNA PCR, Polyadenylation and cDNA synthesis kit (Exiqon, Woburn, MA, USA). cDNA was diluted 20 fold and assayed in 10 ul PCR reactions according to the protocol for miRCURY LNA Universal RT microRNA PCR against a panel of 4 miRNAs and a spike-in control for cDNA synthesis. Of each sample pair from a single subject, the sample with the higher yield based on reverse transcription quantitative polymerase chain reaction (RT-qPCR) was subjected to a scaled up cDNA synthesis and was assayed once by RT-qPCR on the microRNA Ready-to-Use PCR, Human panel I (Exiqon), which includes 372 miRNA primer sets. Negative controls, excluding template from the reverse transcription reaction, were tested and profiled like the samples with individual primer pairs. The amplification was performed in an Applied Biosystems Viia 7 RT-qPCR System (Life Technologies, Carlsbad, CA, USA) in 384 well plates. The amplification curves were analyzed for Ct values using the built-in software, with a single baseline and threshold set manually for each plate. Results with miRNAs shown to be differentially expressed in the initial screen were corroborated using a similar RT-PCR assay minimally in duplicate (Exiqon).

### miRNA data analysis

For RT-qPCR data analysis, 40 miRNAs were selected as standards to normalize Ct values for each plate. These references were chosen because they were among a large subgroup of miRNAs expressed in all samples. We used the delta delta Ct method to calculate expression values. All Ct values were imported into the Rank Product program, which ranks levels for each miRNA within a sample, multiplies the values for all samples in one group to get the rank product, and then calculates a combined probability of the distribution for each RNA in the two groups to determine the probability of differential expression [29, 30]. A cut off for the percentage (proportion) of false positives of 0.05 is taken as significant for differential expression. TCGA RNA-seq data for 314 OSCC samples and 30 controls, were downloaded form the TCGA Data Portal (https://tcga-data.nci.nih.gov/tcga) as normalized miRNA quantification files along with accompanying patient clinical information files. The exact names for TCGA-derived miRNAs were obtained by examining a subset of 5 individual sample isoform quantification files to identify each differentially expressed miRNA based on its mapped genomic site as the 3p or 5p isoform. When ambiguous this designation was not given. Normalized miRNA level counts were loaded directly into the RankProdit Program to perform rank products analysis [29, 30]. Nonnegative Matrix Factorization Consensus Clustering was used to identify the optimal number of distinct samples clusters among the 305 OSCCs in the TCGA dataset with known smoking status accessed through the GenePattern portal www.broadinstitute.org/. It was used to identify the optimal number of distinct samples clusters among the 305 OSCCs in the TCGA dataset [31–33]. First, all miRNAs with more than 50% samples with zero values were filtered out. Clustering was then done based on the levels of the 238 miRNAs which showed the greatest variation in expression levels (normalized standard deviation > 1). The cophenetic coefficient derived served as a measure of correlation between the sample distance induced by the consensus matrix.

Heat maps for visual presentation of the miRNA expression data were generated using BRB Array tools [34]. For the representation of RNA from brush cytology data set we used hierarchical clustering with 1- correlation and average linkage of the expression levels of 50 miRNAs shown to be differentially expressed between OSCC and nonOSCCs based on class comparison using BRB Array tools. For the representation of TCGA expression data nonnegative matrix factorization was used to cluster samples based on the expression levels of 228 most variably expressed miRNAs.

## Results

### miRNA expression in OSCC in ever smokers

This work uses RNA from oral mucosal cells obtained by brush cytology [27, 35] so we first sought to verify this approach to measure miRNA levels by examining miRNAs associated with OSCC in tobacco users. To focus on malignancy-specific pathologic changes, we compared expression of miRNAs in OSCCs of ever smokers versus that in nonmalignant lesions in a similar population. We compared epithelial miRNA from OSCC lesions in 8 patients, and nonmalignant oral lesions/conditions of 9 tobacco users, as outlined in Table 1. These included a granular cell tumor, mucosal aberrations such as fibrous hyperplasia and hyperkeratosis, and a soft tissue ameloblastoma of the gingiva. These lesions often show increased cell proliferation and possibly inflammation, but not other properties such as blocks to apoptosis and the increased tissue invasion that can occur with malignancy. We used the rank product methodology, a nonparametric statistical tool, to determine differentially expressed miRNAs [29, 30]. This test for miRNA differential expression with OSCC in ever smokers revealed one miRNA that was induced specifically with OSCC using the criteria of >2-fold change and at p < 0.05 for the rank product test (Table 2A). This induced miRNA was miR-196a-5p, a miRNA associated with oral cancer in many studies. No miRNAs showed a decrease in expression.

**Table 1:** Ever Smoker Patient data.

**Sample**	**Site** ^a^	**Sex**	**Age**	**Tobacco/Betel** ^b^	**Ethanol** ^c^	**Path. Stage**	**Grade** ^d^	**Immune State**
OSCC110	T	M	50	Bet+Tob	None	T2N0M0	Well Diff	Norm
OSCC129	LG	M	52	F-Tob	L/M	T1N0M0	Well Diff	Norm
OSCC329	LG	M	59	F-Tob	None	T4aN0M0	Poorly Diff	Norm
OSCC359	T	M	61	Tob	Heavy	T4N2cM0	Mod Diff	Norm
OSCC416	T	M	61	Tob	L/M	TcisN0M0	N/A	Norm
OSCC449	LG	F	62	Tob	None	T4NoM0	Mod Diff	Norm
OSCC466	T	M	64	Tob	Heavy	T2N0M0	Poorly Diff	HIV positive
OSCC485	FOM	M	56	Tob	None	T4aN2bM0	Mod Diff	Norm
**Sample**	**Site**	**Sex**	**Age**	**Tobacco/Betel**	**Ethanol**	**Path. Finding**		**Immune State**
BL117	LM	F	46	Tob	L/M	Canicular Adenoma		Norm
BL129	Bu	M	27	Tob	None	Ameloblastoma		Norm
BL149	LG	F	54	F-Tob	None	Hyperkeratosis		Norm
BL319	T	F	33	Tob	L/M	Granular Cell Tumor		Norm
BL367	Bu	M	44	ST	None	Hyperkeratosis, Mucositis		Norm
BL474	LG	F	77	F-Tob	L/M	Fibrous hyperplasia		Norm
BL482	T	M	58	Tob	L/M	Hyperkeratosis		Norm
BL490	UG	M	51	Tob	L/M	Fibrous Hyperplasia		Norm
BL495	UG	M	60	Tob	None	Hyperkeratosis		Norm

^a^T, tongue; LG/UG lower/upper gingiva; FOM, floor of mouth; Bu, buccal mucosa, LM, lip mucosa.

^b^Tob, Tobacco user; if former user than F-Tob.; Bet, betel use; ST, smokeless tobacco user

^c^ L/M, light to moderate intake of ethanol up to 50 grams per day, Heavy, intake of ethanol > 50 g/day.

^d^ well differentiated, Well Diff; moderately differentiated, Mod Diff; Poorly differentiated, Poorly Diff

**Table 2:** MicroRNAs enriched in OSCC and benign oral lesions.

**A. Ever smoker OSCC versus ever smoker benign pathology**			
MicroRNA	Fold induction with OSCC	P Value^b^	Pfp^b^
miR-196a-5p	8.7	0.000104	0.008
miR-144-3p^a^	7.9	0.000104	0.01
**B. Ever smoker OSCC versus never smoker normal**			
MicroRNA	Fold induction with OSCC	P Value	Pfp
miR-196a-5p	61.0	0.000	0.000
miR-144-3p^a^	103.9	0.000	0.000
miR-451a^a^	92.9	0.000	0.000
miR-10b-5p	32.8	7.81E-05	0.006
miR-31-5p	16.1	0.000234	0.015
**C. Never smoker OSCC versus never smoker normal**			
MicroRNA	Fold induction with OSCC	P Value	Pfp
miR-196a-5p	19.2	2.60E-05	0.005
miR-10b-5p	15.0	2.60E-05	0.010
miR-503-5p	15.0	0.000156	0.012
miR-451a^a^	11.7	0.000391	0.025
miR-144-3p^a^	9.6	0.000859	0.033
miR-187-3p	10.0	0.000859	0.037
miR-31-5p	6.7	0.001250	0.040

^a^ microRNA highly expressed in blood,

^b^ rank product probability

^c^ pfp is for the rank product test: percentage false positive predictions

Many published studies on oral cancer largely focus on betel- or tobacco-associated cancers and compare RNA in tumors versus normal nonpathological tissue. We also compared miRNAs enriched with tobacco associated OSCC versus miRNAs in normal oral tissue. With this approach we saw induction of 8 miRNAs including miR196a-5p, miR-10b-5p, miR-31-5p, miR-451a and miR-144-3p (Table 2B). Of these, besides miR-196a-5p, miR-10b-5p and miR-31-5p have been shown to be enriched in earlier studies of head and neck cancer using surgically obtained whole mucosa, and miR144-5p and miR-451a were shown to be induced in the saliva of those with head and neck cancer [36–38].

### miRNA expression in OSCC in never smokers

Never smoker patient features are summarized in Table 3. There were 12 patients, 7 females and 5 males, with ages ranging from 37 to 90 years, with the average age of 67. The control group of subjects ranged in age from 28 to 77; with an average of 61. There were 6 females and four males. MiRNA levels in these never smoker OSCCs were compared to that in nonpathologic mucosa in never smokers.

**Table 3:** Never Smoker OSCC Patient data.

Sample	Site^a^	Sex	Age	Ethanol^b^	Pathologic Stage	Grade^c^	Immune State
OSCC231	T	M	55	None	T1N0M0	Mod Diff	Norm.
OSCC305	T	M	78	L/M	T1N0M0	Mod Diff	Norm.
OSCC308	T	F	74	L/M	T1N0M0	Mod Diff	Rheumatoi Arthritis^d^
OSCC3553	LG	F	84	None	T1N0M0	Mod Diff	Norm.
OSCC357	T	F	74	None	T4N0M0	Mod Diff	Norm.
OSCC413	T	F	63	None	T3N0M0	Mod Diff	Norm.
OSCC453	Bu	F	37	L/M	T2N0M0	Mod Diff	Norm.
OSCC463	LG	F	69	None	T2N0M0	Mod Diff	Norm.
OSCC4231	LG	M	65	None	T4N0M0	Mod Diff	Renal transplant^e^
OSCC4281	LG	F	58	None	T2N0M0	Mod Diff	Norm.
OSCC4291	T	M	90	None	T3N0M0	Mod Diff	Norm.
OSCC5271	LG	M	54	None	T4N0M0	Mod Diff	Renal transplant^e^

^a^T, tongue; LG, lower gingiva; Bu, buccal mucosa.

^b^ L/M, light to moderate intake of ethanol up to 50 grams per day, Heavy, intake of ethanol > 50 g/day.

^c^ well differentiated, Well Diff; moderately differentiated, Mod Diff; Poorly differentiated, Poorly Diff

^d^ treated with anti-inflammatory and Tumor Necrosis Factor inhibitor, etanercept

^e^ treated with standard post-transplant immunosuppressive medications

**Fig 1:**
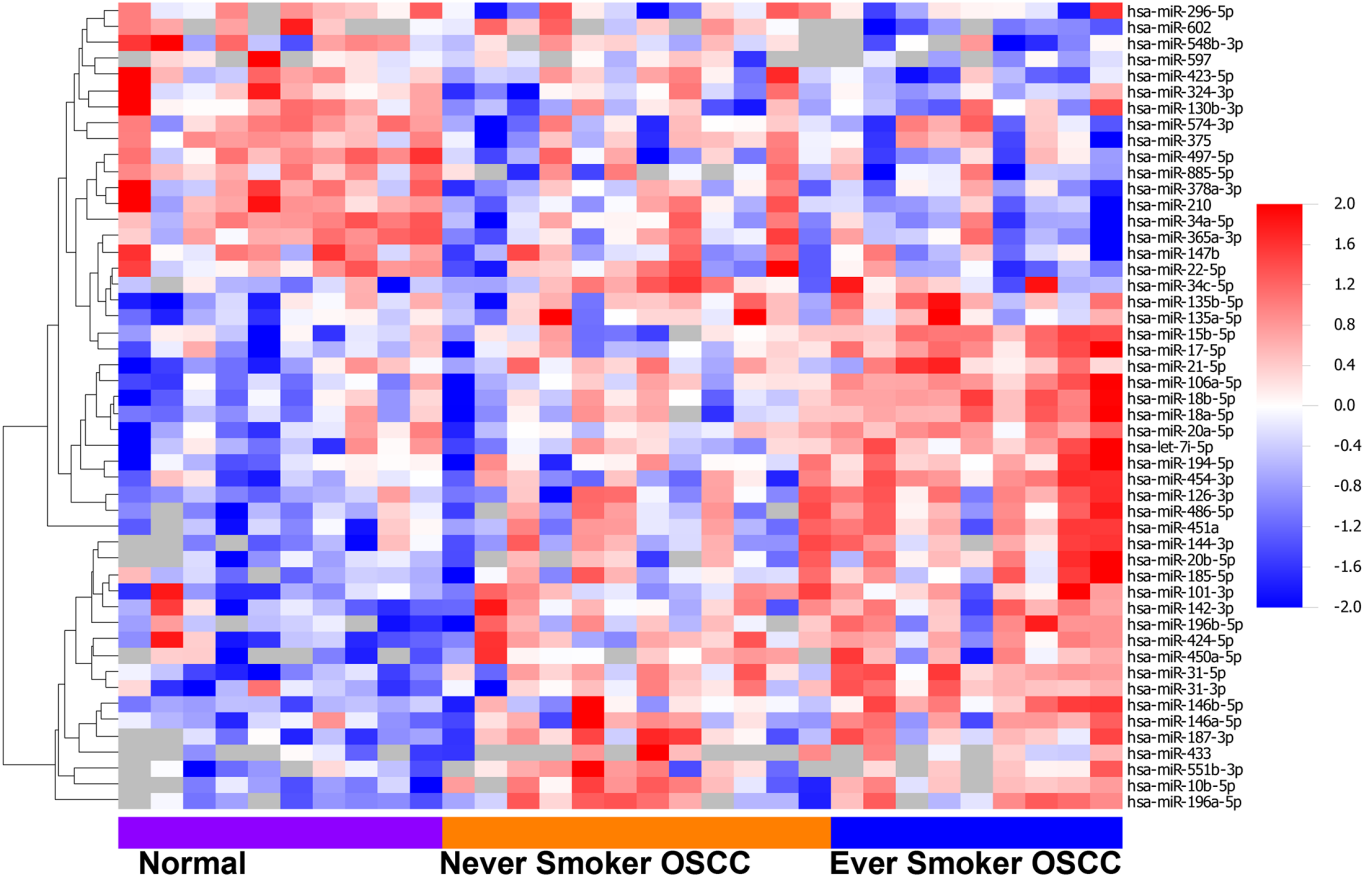
Differentially expressed miRNAs expressed in controls and OSCCs from never smokers and ever smokers. Supervized hierarchical clustering of 50 differentially expressed miRNAs with fold changes above 2, among 9 controls, 12 never smoker OSCCs, and 8 ever smoker OSCCs. Epithelial samples are listed in the columns and miRNAs are in rows. Red indicates higher expression than average and blue indicates lower than average.

Among 372 miRNAs analyzed seven, miR-196a-5p, miR-10b-5p, miR-503-5p, miR-451a, miR-144-3p, miR-187-3p and miR-31-5p, showed increased expression in the OSCC samples of the never smokers, again using the rank product methodology (Table 2c) [29, 30]. Fig 1 shows a heat map with relative expression of the different miRNAs in samples from ever smoker OSCC, never smoker OSCC, and never smoker controls. Again, no miRNAs showed down regulation. This may be because the tumor samples are often a mixture of tumor and normal epithelium while control samples are pure normal epithelium making a loss of expression in tumor hard to detect.

#### Confirmation of similar OSSC miRNA expression changes in ever smokers and never smokers

The TCGA data set of global miRNA expression data of 305 OSCC and control mucosa samples all prepared under standard methods linked to clinical data was used to further explore miRNAs in ever smokers versus never smokers. The set confirmed the observation that ever smokers and never smokers OSCCs show similar miRNA levels. We performed class comparison among the 217 ever smoker OSCC samples versus the 20 ever smoker controls (Table 4a, S5 Table). We then did the same between the 88 never smoker OSCC samples versus the 10 never smoker controls (Table 4b, S6 Table). All 10 miRNAs enriched in the never smoker OSCCs by rank product were contained in the list of miRNAs enriched with the ever smoker group. The ever smoker group showed more miRNAs differentially expressed but that may be due to the larger size of the ever smoker data sets. No miRNAs showed reduced levels in the never smokers tumor though three were depressed in the ever smoker tumor group versus control, miR-375, miR-1-2-3p, and miR-99a (Table 4c). When a direct comparison between ever smoker and never smoker OSCC miRNA expression data was done we saw only one miRNA was differentially expressed, miR-637, confirming how similar the two groups are (Table 4d).

**Table 4:** miRNAs differentially expressed in OSCC and controls in TCGA dataset.

**A. Ever smoker OSCC versus ever smoker normal**			
MicroRNA	Fold induction with OSCC	P Value^a^	Pfp^b^
miR-196b-5p	16.5	0	0
miR-503-5p	8.0	0	0
miR-196a-5p	21.0	0	0
miR-1293	12.1	0	0
miR-196a-5p	10.9	0	0
miR-937-3p	8.5	0	0
miR-210-3p	7.2	0	0
miR-31	11.1	0	0
miR-105-2	35.4	0	0
miR-1269a	52.7	0	0
miR-767	29.3	0	0
miR455	6.2	9.56E-06	0.000714
miR-615-3p	6.7	9.56E-06	0.000769
miR-105-1	25.9	9.56E-06	0.000833
miR-548f	14.0	0.000153	0.010667
miR-1910	7.8	0.000315	0.020625
miR-3648	4.7	0.000478	0.029412
miR-4326	4.9	0.000602	0.035000
miR-224	4.8	0.000746	0.041053
**B. Never smoker OSCC versus never smoker normal**			
MicroRNA	Fold induction with OSCC	P Value	Pfp
miR-105-1	75.8	0	0
miR-105-2	87.3	0	0
miR-196a-5p	11.1	0	0
miR-196b-5p	16.4	0	0
miR-210-3p	5.1	0	0
miR-503-5p	9.2	0	0
miR-767	69.5	3.82E-05	0.00571
miR-31	4.9	4.78E-05	0.00625
miR-1269a	14.5	5.73E-05	0.00667
miR-455	6.7	0.000421	0.04400
**C. Depressed in ever smoker OSCC versus ever smoker normal**			
MicroRNA	Fold induction with Normal	P Value	Pfp
miR-375	0.085	0	0
miR-1-3p	0.14	6.69E-05	0.0175
miR-99a	0.23	0.000182	0.038
**D. Ever smoker OSCC versus never smoker OSCC**			
MicroRNA	Fold induction with ever smokers	P Value	Pfp
miR-637	3.4	0	0

^a^ rank product probability

^b^ pfp is for the rank product test: percentage false positive predictions

**Fig 2:**
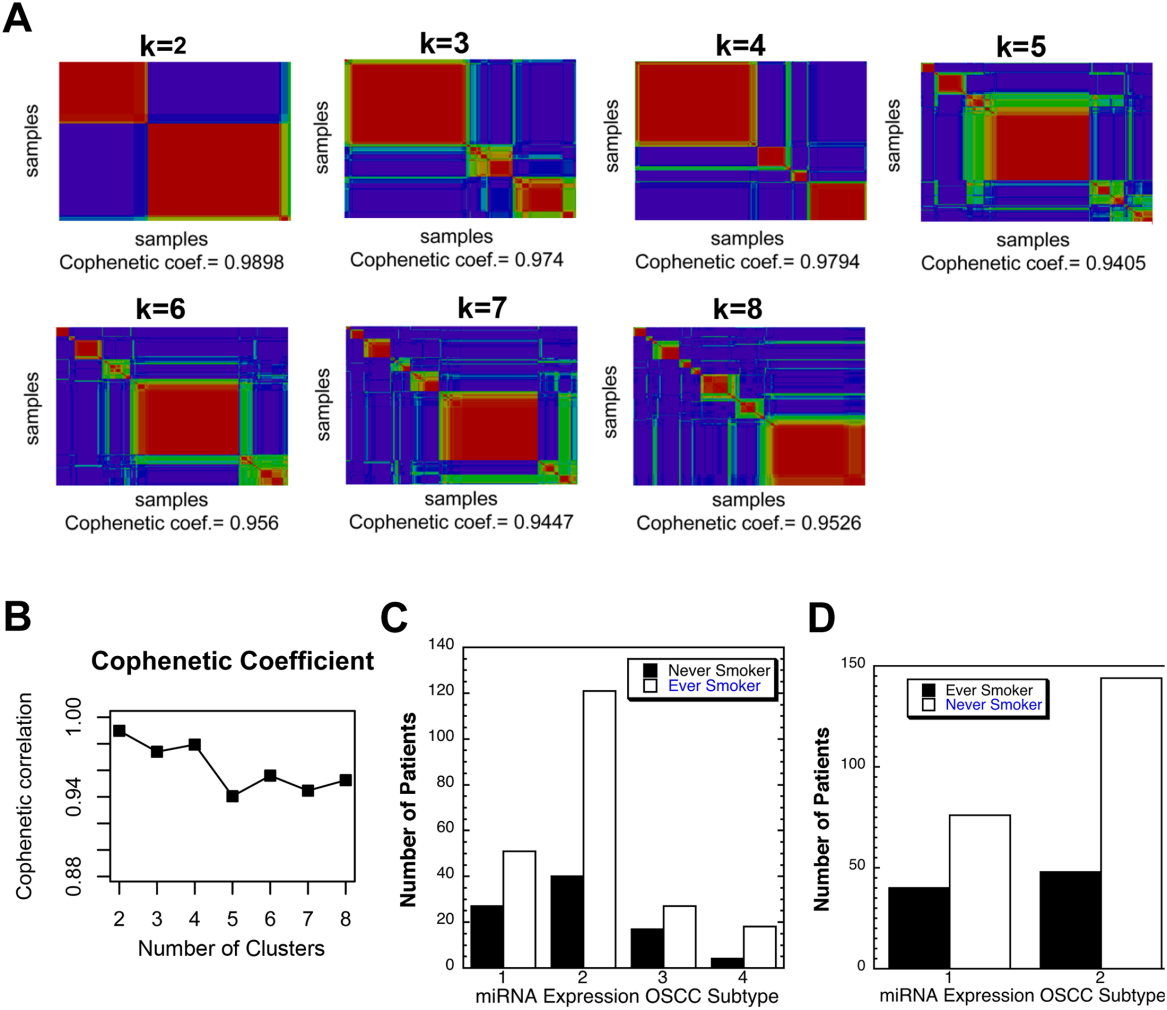
Discovery of OSCC subtypes based on miRNA expression among 305 OSCCs in the TCGA dataset. A) Nonnegatiive matrix factorization consensus clustering showed support for two and four OSCC subtypes based on the maximal cophenetic value at K = 2 and 4. B) Consensus matrices for the samples were calculated at k = 2, 3, 4, 5, 6, 7, 8. A comparison of the visualized consensus matrices allowed a visual assessment of the best rank. Clear block patterns along the diagonal of the consensus matrices indicated robustness of the clustering. C) Analysis of the ever smokers and never smokers in both miRNA based subclass revealed a lack of a difference in the proportions of the two risk groups based on smoking experience, p < 0.059 by Fisher Exact Test D) Analysis of the ever smokers and never smokers in each of 4 miRNA based subclasses revealed no significant difference in proportions of ever and never smokers in any group.

It was shown some time ago that HNSCC fall into 4 subtypes based on global miRNA expression, though one of the groups is heavily weighted to oral pharyngeal tumors [39–41]. We performed unsupervised clustering using nonnegative matrix factorization (NMF), which identifies common gene/RNA expression patterns, or metagenes, among samples [31]. It calculates a cophenetic coefficient for each value of K (number of clusters) which is maximal when the clusters show maximal separation. Using the 238 miRNAs most variably expressed among the 305 OSCC samples K = 2–7 produced the highest cophenetic coefficient at K = 2 and 4 indicating two or four clusters of samples (Fig 2) [32]. A heat map reveas differential expression of a subset of the miRNAs used to separate the cases into two subtypes or clusters (S2 Fig). Given these two OSCC groups, we examined tobacco usage among the subjects and found it was possible one group showed a slightly higher number of smokers but this did not reach significance (Fig 2C). When we compared pack year exposure between these two groups we found the first group showed almost 40% lower exposure 62 ≠2.6 pack years versus the second 100+4.4 with t< 0.036. Suggesting tobacco exposure may indeed have some effect on miRNA expression.

When 4 OSCC subclasses were examined for tobacco usage there was no significant difference in proportion of ever and never smokers based on the Chi Square test though some groups were small making statistical analysis difficult (Fig 2A and 2D).

## Discussion

The etiology of OSCC of never smokers is unknown. If OSCC in never smokers is a distinct subtype of this cancer then regulatory RNAs in the tumor epithelium may be different than those of tobacco-associated OSCCs. Tumors from both cohorts are generally not associated with p16 enrichment or transforming HPV gene expression and that was the case here with one subject positive for p16 and one positive for HPV16 RNA (S2 Table) [24, 26]. This study sought to examine OSCC epithelium to discern miRNA misexpression with this disease in never smokers. We measured miRNAs enriched in OSCC epithelium of never smokers and in ever smokers both compared to normal tissue. We found that miR-144-3p, miR-451a, miR-10b-5p, mir-31-5p, and miR-196a-5p were enriched with tumor formation whether the subject was an ever smoker or never smoker. Examination of the TCGA OSCCs and miRNA expression also suggests that overall there was much alike in OSCCs from ever and never smokers (S3 Table).

It was possible tobacco played a role in changing miRNA expression in a subgroup of OSCCs; however it has been noted that the “classical” subtype of HNSCCs, associated with heavy tobacco usage, is only a minor component of OSCCs. Indeed as published the TCGA group showed only 11.2% of group of 178 oral tumors showed the “classical” mRNA expression profile [39]. When we separated the OSCCs based on miRNA expression we found two or four subclasses (Fig 2). The two subclass clusters showed the maximal agreement with the mRNA based subclassifications with group one having most of the “atypical” mRNA expression pattern and group two having most of the “classical pattern” [40, 41]. Curiously group two of the two subclasses showed a nominally higher percentage of smokers that did not reach statistical significance, but there was a 40% increase in cigarette pack year exposure 62±2.6 versus 100±4.4, t < 0.0389. These findings show tobacco usage may have some effect on miRNA expression, but other unknown factors have larger effects, and that relying on tobacco exposure to assign tumor type is not wise.

This study in part focuses on one cell type, epithelium, obtained by brush cytology, making it more sensitive to changes in expression of miRNAs found mainly in this specific cell type [27]. MiRNAs enriched in stroma and not epithlieum of tumors would not be apparent. It is important to note that anything that causes the brush to acquire cells normally not present in the epithelium, such as the epithelial invasion of lymphocytes that can occur in inflamed OSCC, can also result in changes in RNAs in the sample. Some samples may also have blood contamination while others do not. In particular, malignant lesions can be more highly vascularized with blood vessels next to epithelial cells, which can greatly increase the mix of blood cells exposed to the brush. We saw elevated levels of miR-451a in both tumor groups compared to normal tissue. This RNA is highly expressed in red blood cells (RBCs) and is a well-known indicator of RBC RNA contamination in plasma samples [42, 43]. MiR-451a levels were highest in OSCC samples of ever smokers and lowest in normal samples of never smokers. In the samples, levels of miR-451a correlated with a second blood-linked miRNA, miR-144-3p, with a correlation coefficient of 0.91 [36, 37, 44], data not shown. While we showed both miR-451a and miR-144-3p are markers for OSCC, this property is almost certainly indirect and there is little reason to believe that expression of these two miRNAs is elevated in OSCC epithelial cells but that instead the tumors tend to have blood vessels in the epithelium. The TCGA dataset which contains RNA from surgically obtained OSCC and control tissue that is mainly epithelium and less than 50% stroma did not show increases of these miRNAs, probably because all samples had some blood.

In a study of lingual squamous cell carcinoma in young never smoker patients there was little evidence for differences in mutation spectrum in OSCCs of that group versus OSCCs of old smokers [45]. In contrast one HNSCC type, laryngeal tumors from tobacco users, showed a mutation spectrum, with a decrease of c>t mutations and an increase of c>a mutations, similar to lung carcinoma [45, 46]. Both of these cancers are believed to be initiated by the mutagenic polycyclic aromatic hydrocarbons in tobacco and their combustion products. Because tobacco usage did not correlate with known tobacco mutation spectrums one might speculate that among many ideas, It may increase mutation rate via interactions with other compounds, or oral micro-organisms may change the metabolism of tobacco chemicals, so that the types of mutations that are induced change. Alternatively as Pickering et al. suggest tobacco may only be a tumor promoter for the cancer process in the mouth [45]. It could do this by increasing inflammation in oral tissue, or cell proliferation, decreasing apoptosis, etc. Our finding that miRNA expression was quite similar in OSCCs of ever and never smokers suggests tumor formation may be through similar processes in both groups. Although it would seem tobacco usage increases the probability of OSCC formation without modifying the process very much, how it does this remains a mystery.

The study of miRNA in OSCC epithelium of never and ever smokers revealed three miRNAs that are enriched in epithelial OSCC samples obtained by brush cytology and the samples obtained surgically in the TCGA project (Table 5). Two of these miRNAs have been shown to be enriched with OSCC in earlier studies, miR-196a-5p and miR31-5p. MiR-31-5p has been shown to be induced in HNSCC and OSCC compared to normal mucosa and is also induced in the epithelium with several inflammatory disorders like Idiopathic Bowel Syndrome [34–36]. Curiously, this miRNA was induced in benign lesions versus normal controls suggesting its induction is not specific to malignancy (S3 Table) and in a recent study was shown to be induced with leukoplakia [47]. MiR-503-5p was found to be induced with both TCGA OSCC groups and the nonsmoker OSCC sampled by brush cytology. It has been shown to be depressed in some tumor types, though in esophagus, colon, adrenocortical and parathyroid tumors it is enriched in advanced stages [48–51] [52]. Finally, miR-196a-5p was induced in all OSCC groups studied, suggesting it may be part of a key step in oral carcinogenesis (Table 2 and Fig 2). As it was not induced in the epithelium of nonmalignant oral pathologies, but only in OSCCs, it also may serve as an aid to diagnosis of OSCC (S3 Table). MiR-196a-5p has been shown to be induced in two studies of OSCC recently and in laryngeal carcinoma, and there is good evidence for a functional role in cervical squamous cell carcinoma [53–57]. Studies in cervical cancer suggest it has a role in regulating cell proliferation and that it targets p27, FOXO1 and the (PI3K) AKT pathway, and HOXC8, a cell remodeling protein [53, 56] [53, 58]. Additional studies in laryngeal cancer types saw properties for this alleged oncomir in cell migration and proliferation [55]. It seems to be highly induced in a large proportion of OSCCs of tobacco users, nontobacco users and betel nut users [53, 56, 58].

**Table 5:** MiRNAs enriched with OSCC in RNA from brush cytology or surgically obtained samples.

Cytology Sample Data		TCGA Surgical Sample Data	
Ever smoker OSCC VS Normal/Benign pathology	Never smoker OSCC VS Normal	Ever smoker OSCC VS Normal	Never smoker OSCC VS Normal
**miR-196a-5p**	**miR-196a-5p**	**miR-196b-5p**	**miR-105-1**
**miR-144-3p**	**miR-10b-5p**	**miR-503-5p**	**miR-105-2**
**miR-451a**	miR-503-5p	**miR-196a-1-5p**	**miR-196a-1-5p**
**miR-10b-5p**	**miR-451a**	miR-1293^a^	**miR-196b-5p**
**miR-31-5p**	**miR-144-3p**	miR-196a-2-5p	**miR-210**
	miR-187-3p	miR-937^a^-3p	**miR-503-5p**
	**miR-31-5p**	**miR-210-3p**	**miR-767**
		**miR-31**	**miR-31**
		**miR-105-2**	**miR-1269**
		**miR-1269** ^a^	**miR-455**
		**miR-767** ^a^	
		**miR-455**	
		miR-615-3p	
		**miR-105-1**	
		miR-548f-1^a^	
		miR-1910^a^	
		miR-3648^a^	
		miR-4326^a^	
		miR-224	

^a^ miRNAs which are not included in the cytology RNA screens; miRNAs in bold are enriched in both ever smokers and never smokers OSCCs

The work described suggests that there is much alike in OSCC in never and ever tobacco users in regard to miRNA misexpression so we sought to determine if these OSCC patients may share other factors related to OSCC causation. In addition to tobacco exposure, high ethanol intake and immunosuppression represent additional risk factors that have been linked to increased head and neck and OSCC [59–61]. Based on ethanol consumption the two groups were different. The never smokers showed statistically significant lower rates of total ethanol consumption in the TCGA data set and much lower number of subjects who consumed ethanol (S4 Table). This was supported by the current study where half of the ever smokers consumed ethanol regularly, two at heavy levels, while only 3 out of 12 of the never smokers reported any ethanol usage (Tables 1 and 2). This difference did not reach statistical significance. While immunoupression was not reported for TCGA patients, in the present study 2 of 12 never smoker patients were renal transplant patients treated with immuno-suppressants, with one additional patient with rheumtatoid arthritis treated with an anti- tumor necrosis factor drug, etanerecept [60]. No Immune suppression treatment history was found for the ever smoker OSCC group, though one subject was positive for HIV. In conclusion, despite the differences in risk factors between the two OSCC groups with different events presumably causing the cancers, the OSCC miRNA profile for ever smokers and never smokers was similar suggesting there is limited variability in miRNA changes when a normal cell progresses to OSCC. More work will be required to discern just how similar the carcinogenesis process is in the two groups and in other high-risk groups such as betel nut and ethanol users.

## Supporting Information

S1 Table Sequences of primers and probe used to amplify and detect HPV16 E6 mRNA in the mRNA samples from never smoker OSCC lesions. (DOC)

S2 Table Never Smoker OSCC tumors tested for p16 and HPV16 E6 presence. (DOC)

S3 Table MicroRNAs enriched in benign oral lesions of ever smokers versus never smokers normal. (DOC)

S4 Table Ethanol Usage of Patients with OSCC in TCGA data set. (DOC)

S5 Table Rank product test of differential miRNA expression of ever smoker OSCC versus ever smoker normal epithelium in TCGA data set. (XLSX)

S6 Table Rank product test of differential miRNA expression of never smoker OSCC versus never smoker normal epithelium in TCGA data set. (XLSX)

S1 Fig Comparison of miRNA expression profile from 1x and 9x dilution of a sample processed with Trizol and RNeasy chromatography shows no change. Half of a single brush oral mucosal sample was diluted 9x in Trizol and then both halves were subjected to RT-PCR to quantify 13 different miRNAs. We show that for the methodology used, storage of the sample frozen in Trizol, followed by 1-bromo-3-chloropropane (BCP) phase separation, then immediate glass filter binding using RNeasy columns (Qiagen), the range of miRNA species recovered was uniform from a single sample. This occurred whether the same was concentrated or diluted 9x. MiRNA from the concentrated and diluted samples was converted to cDNA then quantified using RT-PCR. A comparison of Ct values for 13 detectable miRNA species revealed similar relative amounts of each species with a correlation coefficient 0.96.
(DOC)

S2 Fig Heatmap of Nonnegative Matrix Factorization based clustering of 305 OSCC from the TCGA dataset based on miRNA expression reveals two subtypes. Sample clusters are identified by the colored horizontal bar. The color key provides information on relative expression levels. The relative expression levels of 50 variable miRNAs are shown.
(PDF)

## References

[1] Ambros V. The functions of animal microRNAs. Nature. 2004;431(7006):350–5. Epub 2004/09/17. 15372042 10.1038/nature02871

[2] Esquela-Kerscher A, Slack FJ. Oncomirs—microRNAs with a role in cancer. Nature reviews Cancer. 2006;6(4):259–69. Epub 2006/03/25. 16557279 10.1038/nrc1840

[3] Croce CM, Calin GA. miRNAs, cancer, and stem cell division. Cell. 2005;122(1):6–7. Epub 2005/07/13. 16009126 10.1016/j.cell.2005.06.036

[4] Blenkiron C, Miska EA. miRNAs in cancer: approaches, aetiology, diagnostics and therapy. Human molecular genetics. 2007;16 Spec No 1:R106–13. Epub 2007/09/26. 17613543 10.1093/hmg/ddm056

[5] Calin GA, Croce CM. MicroRNA signatures in human cancers. Nature reviews Cancer. 2006;6(11):857–66. Epub 2006/10/25. 17060945 10.1038/nrc1997

[6] Lu J, Getz G, Miska EA, Alvarez-Saavedra E, Lamb J, Peck D, et al MicroRNA expression profiles classify human cancers. Nature. 2005;435(7043):834–8. Epub 2005/06/10. 15944708 10.1038/nature03702

[7] Di Leva G, Garofalo M, Croce CM. MicroRNAs in cancer. Annual review of pathology. 2014;9:287–314. Epub 2013/10/02. 10.1146/annurev-pathol-012513-104715 24079833 PMC4009396

[8] Tay FC, Lim JK, Zhu H, Hin LC, Wang S. Using artificial microRNA sponges to achieve microRNA loss-of-function in cancer cells. Advanced drug delivery reviews. 2014. Epub 2014/05/27. 10.1016/j.addr.2014.05.010 24859534

[9] Hansen TB, Jensen TI, Clausen BH, Bramsen JB, Finsen B, Damgaard CK, et al Natural RNA circles function as efficient microRNA sponges. Nature. 2013;495(7441):384–8. Epub 2013/03/01. 10.1038/nature11993 23446346

[10] Wu BH, Xiong XP, Jia J, Zhang WF. MicroRNAs: new actors in the oral cancer scene. Oral oncology. 2011;47(5):314–9. Epub 2011/04/09. 10.1016/j.oraloncology.2011.03.019 21474366

[11] Chen D, Cabay RJ, Jin Y, Wang A, Lu Y, Shah-Khan M, et al MicroRNA Deregulations in Head and Neck Squamous Cell Carcinomas. Journal of oral & maxillofacial research. 2013;4(1):e2. Epub 2014/01/15. 10.5037/jomr.2013.4102 PMC3886106 24422025

[12] Hui AB, Lenarduzzi M, Krushel T, Waldron L, Pintilie M, Shi W, et al Comprehensive MicroRNA profiling for head and neck squamous cell carcinomas. Clinical cancer research: an official journal of the American Association for Cancer Research. 2010;16(4):1129–39. Epub 2010/02/11. 20145181 10.1158/1078-0432.CCR-09-2166

[13] John K, Wu J, Lee BW, Farah CS. MicroRNAs in Head and Neck Cancer. International journal of dentistry. 2013;2013:650218 Epub 2013/11/22. 10.1155/2013/650218 24260035 PMC3821954

[14] Varghese AM, Zakowski MF, Yu HA, Won HH, Riely GJ, Krug LM, et al Small-cell lung cancers in patients who never smoked cigarettes. Journal of thoracic oncology: official publication of the International Association for the Study of Lung Cancer. 2014;9(6):892–6. Epub 2014/05/16. 10.1097/JTO.0000000000000142 PMC4199745 24828667

[15] Mendez E. Biomarkers of HPV infection in oropharyngeal carcinomas: can we find simplicity in the puzzle of complexity? Cancer research. 2012;72(19):4896–8. Epub 2012/09/20. 10.1158/0008-5472.CAN-12-3285 22991303

[16] Westra WH. Detection of human papillomavirus (HPV) in clinical samples: Evolving methods and strategies for the accurate determination of HPV status of head and neck carcinomas. Oral oncology. 2014;50(9):771–9. Epub 2014/06/17. 10.1016/j.oraloncology.2014.05.004 24932529 PMC4318232

[17] Kruger M, Pabst AM, Walter C, Sagheb K, Gunther C, Blatt S, et al The prevalence of human papilloma virus (HPV) infections in oral squamous cell carcinomas: A retrospective analysis of 88 patients and literature overview. Journal of cranio-maxillo-facial surgery: official publication of the European Association for Cranio-Maxillo-Facial Surgery. 2014. Epub 2014/06/21. 10.1016/j.jcms.2014.04.022 24947612

[18] Durr ML, Li D, Wang SJ. Oral cavity squamous cell carcinoma in never smokers: analysis of clinicopathologic characteristics and survival. American journal of otolaryngology. 2013;34(5):388–93. Epub 2013/04/02. 10.1016/j.amjoto.2013.01.017 23540889

[19] Farshadpour F, Roepman P, Hordijk GJ, Koole R, Slootweg PJ. A gene expression profile for non-smoking and non-drinking patients with head and neck cancer. Oral diseases. 2012;18(2):178–83. Epub 2011/11/01. 10.1111/j.1601-0825.2011.01861.x 22035108

[20] Koch WM, Lango M, Sewell D, Zahurak M, Sidransky D. Head and neck cancer in nonsmokers: a distinct clinical and molecular entity. The Laryngoscope. 1999;109(10):1544–51. Epub 1999/10/16. 10522920 10.1097/00005537-199910000-00002

[21] Heaton CM, Durr ML, Tetsu O, van Zante A, Wang SJ. TP53 and CDKN2a mutations in never-smoker oral tongue squamous cell carcinoma. The Laryngoscope. 2014;124(7):E267–73. Epub 2014/01/17. 10.1002/lary.24595 24431303

[22] Sebastian P, Babu JM, Prathibha R, Hariharan R, Pillai MR. Anterior tongue cancer with no history of tobacco and alcohol use may be a distinct molecular and clinical entity. Journal of oral pathology & medicine: official publication of the International Association of Oral Pathologists and the American Academy of Oral Pathology. 2014;43(8):593–9. Epub 2014/05/09. 10.1111/jop.12175 24809775

[23] Braakhuis BJ, Snijders PJ, Keune WJ, Meijer CJ, Ruijter-Schippers HJ, Leemans CR, et al Genetic patterns in head and neck cancers that contain or lack transcriptionally active human papillomavirus. Journal of the National Cancer Institute. 2004;96(13):998–1006. Epub 2004/07/09. 15240783 10.1093/jnci/djh183

[24] Li R, Faden DL, Fakhry C, Langelier C, Jiao Y, Wang Y, et al Clinical, genomic, and metagenomic characterization of oral tongue squamous cell carcinoma in patients who do not smoke. Head & neck. 2014. Epub 2014/06/24. 10.1002/hed.23807 PMC4272912 24954188

[25] Lingen MW, Xiao W, Schmitt A, Jiang B, Pickard R, Kreinbrink P, et al Low etiologic fraction for high-risk human papillomavirus in oral cavity squamous cell carcinomas. Oral oncology. 2013;49(1):1–8. Epub 2012/07/31. 10.1016/j.oraloncology.2012.07.002 22841678

[26] Poling JS, Ma XJ, Bui S, Luo Y, Li R, Koch WM, et al Human papillomavirus (HPV) status of non-tobacco related squamous cell carcinomas of the lateral tongue. Oral oncology. 2014;50(4):306–10. Epub 2014/02/04. 10.1016/j.oraloncology.2014.01.006 24485566 PMC3972491

[27] Schwartz JL, Panda S, Beam C, Bach LE, Adami GR. RNA from brush oral cytology to measure squamous cell carcinoma gene expression. Journal of oral pathology & medicine: official publication of the International Association of Oral Pathologists and the American Academy of Oral Pathology. 2008;37(2):70–7. Epub 2008/01/17. 10.1111/j.1600-0714.2007.00596.x 18197850

[28] Kim YK, Yeo J, Kim B, Ha M, Kim VN. Short structured RNAs with low GC content are selectively lost during extraction from a small number of cells. Molecular cell. 2012;46(6):893–5. Epub 2012/07/04. 10.1016/j.molcel.2012.05.036 22749402

[29] Laing E, Smith CP. RankProdIt: A web-interactive Rank Products analysis tool. BMC research notes. 2010;3:221 Epub 2010/08/10. 10.1186/1756-0500-3-221 20691047 PMC2930644

[30] Hong F, Breitling R, McEntee CW, Wittner BS, Nemhauser JL, Chory J. RankProd: a bioconductor package for detecting differentially expressed genes in meta-analysis. Bioinformatics. 2006;22(22):2825–7. Epub 2006/09/20. 16982708 10.1093/bioinformatics/btl476

[31] Brunet JP, Tamayo P, Golub TR, Mesirov JP. Metagenes and molecular pattern discovery using matrix factorization. Proceedings of the National Academy of Sciences of the United States of America. 2004;101(12):4164–9. Epub 2004/03/16. 15016911 10.1073/pnas.0308531101 PMC384712

[32] Monti S, Tamayo P, Mesirov JP, Golub TR. Consensus clustering: A resampling-based method for class discovery and visualization of gene expression microarray data. Machine Learning. 2003;52:91–118.

[33] Reich M, Liefeld T, Gould J, Lerner J, Tamayo P, Mesirov JP. GenePattern 2.0. Nature genetics. 2006;38(5):500–1. Epub 2006/04/28. 16642009 10.1038/ng0506-500

[34] Simon R, Lam A, Li MC, Ngan M, Menenzes S, Zhao Y. Analysis of gene expression data using BRB-ArrayTools. Cancer informatics. 2007;3:11–7. Epub 2007/01/01. 19455231 PMC2675854

[35] Kolokythas A, Schwartz JL, Pytynia KB, Panda S, Yao M, Homann B, et al Analysis of RNA from brush cytology detects changes in B2M, CYP1B1 and KRT17 levels with OSCC in tobacco users. Oral oncology. 2011;47(6):532–6. Epub 2011/05/10. 10.1016/j.oraloncology.2011.03.029 21549635

[36] Lu YC, Chen YJ, Wang HM, Tsai CY, Chen WH, Huang YC, et al Oncogenic function and early detection potential of miRNA-10b in oral cancer as identified by microRNA profiling. Cancer Prev Res (Phila). 2012;5(4):665–74. Epub 2012/02/10. 22318752 10.1158/1940-6207.CAPR-11-0358

[37] Tu HF, Lin SC, Chang KW. MicroRNA aberrances in head and neck cancer: pathogenetic and clinical significance. Current opinion in otolaryngology & head and neck surgery. 2013;21(2):104–11. Epub 2013/01/24. 23340306 10.1097/MOO.0b013e32835e1d6e

[38] Xie Z, Chen G, Zhang X, Li D, Huang J, Yang C, et al Salivary microRNAs as promising biomarkers for detection of esophageal cancer. PloS one. 2013;8(4):e57502 Epub 2013/04/06. 10.1371/journal.pone.0057502 23560033 PMC3613402

[39] Comprehensive genomic characterization of head and neck squamous cell carcinomas. Nature. 2015;517(7536):576–82. Epub 2015/01/30. 10.1038/nature14129 25631445 PMC4311405

[40] Chung CH, Parker JS, Karaca G, Wu J, Funkhouser WK, Moore D, et al Molecular classification of head and neck squamous cell carcinomas using patterns of gene expression. Cancer cell. 2004;5(5):489–500. Epub 2004/05/18. 15144956 10.1016/s1535-6108(04)00112-6

[41] Walter V, Yin X, Wilkerson MD, Cabanski CR, Zhao N, Du Y, et al Molecular subtypes in head and neck cancer exhibit distinct patterns of chromosomal gain and loss of canonical cancer genes. PloS one. 2013;8(2):e56823 Epub 2013/03/02. 10.1371/journal.pone.0056823 23451093 PMC3579892

[42] MacLellan SA, MacAulay C, Lam S, Garnis C. Pre-profiling factors influencing serum microRNA levels. BMC clinical pathology. 2014;14:27 Epub 2014/08/06. 10.1186/1472-6890-14-27 25093010 PMC4107491

[43] Pritchard CC, Cheng HH, Tewari M. MicroRNA profiling: approaches and considerations. Nature reviews Genetics. 2012;13(5):358–69. Epub 2012/04/19. 10.1038/nrg3198 22510765 PMC4517822

[44] Severino P, Bruggemann H, Andreghetto FM, Camps C, Klingbeil Mde F, de Pereira WO, et al MicroRNA expression profile in head and neck cancer: HOX-cluster embedded microRNA-196a and microRNA-10b dysregulation implicated in cell proliferation. BMC cancer. 2013;13:533 Epub 2013/11/12. 10.1186/1471-2407-13-533 24209638 PMC3826519

[45] Pickering CR, Zhang J, Neskey DM, Zhao M, Jasser SA, Wang J, et al Squamous cell carcinoma of the oral tongue in young non-smokers is genomically similar to tumors in older smokers. Clinical cancer research: an official journal of the American Association for Cancer Research. 2014;20(14):3842–8. Epub 2014/05/31. 24874835 10.1158/1078-0432.CCR-14-0565 PMC4102633

[46] Alexandrov LB, Nik-Zainal S, Wedge DC, Aparicio SA, Behjati S, Biankin AV, et al Signatures of mutational processes in human cancer. Nature. 2013;500(7463):415–21. Epub 2013/08/16. 10.1038/nature12477 23945592 PMC3776390

[47] De Sarkar N, Roy R, Mitra JK, Ghose S, Chakraborty A, Paul RR, et al A Quest for miRNA Bio-Marker: A Track Back Approach from Gingivo Buccal Cancer to Two Different Types of Precancers. PloS one. 2014;9(8):e104839 Epub 2014/08/16. 10.1371/journal.pone.0104839 25126847 PMC4134240

[48] Corbetta S, Vaira V, Guarnieri V, Scillitani A, Eller-Vainicher C, Ferrero S, et al Differential expression of microRNAs in human parathyroid carcinomas compared with normal parathyroid tissue. Endocrine-related cancer. 2010;17(1):135–46. Epub 2009/11/21. 10.1677/ERC-09-0134 19926710

[49] Lu ZM, Lin YF, Jiang L, Chen LS, Luo XN, Song XH, et al Micro-ribonucleic acid expression profiling and bioinformatic target gene analyses in laryngeal carcinoma. OncoTargets and therapy. 2014;7:525–33. Epub 2014/04/18. 10.2147/OTT.S59871 24741319 PMC3983076

[50] Park YT, Jeong JY, Lee MJ, Kim KI, Kim TH, Kwon YD, et al MicroRNAs overexpressed in ovarian ALDH1-positive cells are associated with chemoresistance. Journal of ovarian research. 2013;6(1):18 Epub 2013/03/26. 10.1186/1757-2215-6-18 23522567 PMC3637599

[51] Tao K, Yang J, Guo Z, Hu Y, Sheng H, Gao H, et al Prognostic value of miR-221-3p, miR-342-3p and miR-491-5p expression in colon cancer. American journal of translational research. 2014;6(4):391–401. Epub 2014/07/31. 25075256 PMC4113501

[52] Ide S, Toiyama Y, Shimura T, Kawamura M, Yasuda H, Saigusa S, et al MicroRNA-503 promotes tumor progression and acts as a novel biomarker for prognosis in oesophageal cancer. Anticancer research. 2015;35(3):1447–51. Epub 2015/03/10. 25750296

[53] Hou T, Ou J, Zhao X, Huang X, Huang Y, Zhang Y. MicroRNA-196a promotes cervical cancer proliferation through the regulation of FOXO1 and p27Kip1. British journal of cancer. 2014;110(5):1260–8. Epub 2014/01/16. 10.1038/bjc.2013.829 24423924 PMC3950858

[54] Liu CJ, Tsai MM, Tu HF, Lui MT, Cheng HW, Lin SC. miR-196a overexpression and miR-196a2 gene polymorphism are prognostic predictors of oral carcinomas. Annals of surgical oncology. 2013;20 Suppl 3:S406–14. Epub 2012/11/10. 10.1245/s10434-012-2618-6 23138850

[55] Saito K, Inagaki K, Kamimoto T, Ito Y, Sugita T, Nakajo S, et al MicroRNA-196a is a putative diagnostic biomarker and therapeutic target for laryngeal cancer. PloS one. 2013;8(8):e71480 Epub 2013/08/24. 10.1371/journal.pone.0071480 23967217 PMC3743786

[56] Villegas-Ruiz V, Juarez-Mendez S, Perez-Gonzalez OA, Arreola H, Paniagua-Garcia L, Parra-Melquiadez M, et al Heterogeneity of microRNAs expression in cervical cancer cells: over-expression of miR-196a. International journal of clinical and experimental pathology. 2014;7(4):1389–401. Epub 2014/05/13. 24817935 PMC4014219

[57] Zhang H, Su YL, Yu H, Qian BY. Meta-Analysis of the Association between Mir-196a-2 Polymorphism and Cancer Susceptibility. Cancer biology & medicine. 2012;9(1):63–72. Epub 2012/03/01. 23691458 10.3969/j.issn.2095-3941.2012.01.012 PMC3643645

[58] Zhang J, Zheng F, Yu G, Yin Y, Lu Q. miR-196a targets netrin 4 and regulates cell proliferation and migration of cervical cancer cells. Biochemical and biophysical research communications. 2013;440(4):582–8. Epub 2013/10/15. 10.1016/j.bbrc.2013.09.142 24120501

[59] Pezzuto F, Buonaguro L, Caponigro F, Ionna F, Starita N, Annunziata C, et al Update on Head and Neck Cancer: Current Knowledge on Epidemiology, Risk Factors, Molecular Features and Novel Therapies. Oncology. 2015;89(3):125–36. Epub 2015/05/15. 10.1159/000381717 25967534

[60] Rabinovics N, Hadar T, Mizrachi A, Bachar G, Purim O, Popovtzer A. Adjuvant treatment for head and neck cancer in solid organ transplant recipients. Oral oncology. 2015;51(5):e23–5. Epub 2015/03/11. 10.1016/j.oraloncology.2015.02.004 25753559

[61] Rhodus NL, Kerr AR, Patel K. Oral cancer: leukoplakia, premalignancy, and squamous cell carcinoma. Dental clinics of North America. 2014;58(2):315–40. Epub 2014/03/25. 10.1016/j.cden.2013.12.004 24655525

