# Supplementary material for: Similar Squamous Cell Carcinoma Epithelium microRNA Expression in Never Smokers and Ever Smokers
Source: PLoS One. 2015 Nov 6;10(11):e0141695. doi: 10.1371/journal.pone.0141695 (PMC4636300; doi:10.1371/journal.pone.0141695)
Supplement: S1 Table — (DOC) [file pone.0141695.s001.doc]

S1 Table Sequences of primers and probe used to amplify and detect HPV16 E6 mRNA in the mRNA samples from never smoker OSCC lesions

External Primers

47: 5’-CCAGAAAGTTACCACAG-3’

469: 5’-GGTTTCTCTACGTGTTC-3’

Internal Primers

297: 5’-CAACAAACCGTTGTGTGATTTG-3’

385: 5’-TTTGTCCAGATGTCTTTGCTT-3’

Probe-342: 5’-/56-FAM/AAAGCCACT/ZEN/GTGTCCTGA-3’
