# Supplementary material for: Similar Squamous Cell Carcinoma Epithelium microRNA Expression in Never Smokers and Ever Smokers
Source: PLoS One. 2015 Nov 6;10(11):e0141695. doi: 10.1371/journal.pone.0141695 (PMC4636300; doi:10.1371/journal.pone.0141695)
Supplement: S3 Table — (DOC) [file pone.0141695.s003.doc]

Supplemental Table 3 MicroRNAs enriched in benign oral lesions

of ever smokers versus normal mucosa of never smokers

| MicroRNA | Fold induction with benign lesion | pfp^b^ |
| --- | --- | --- |
| miR-133a | 67.2 | 0 |
| miR-451a^a^ | 29.4 | 0.0033 |
| miR-10b-5p | 18.5 | 0.0040 |
| miR-144-3p^a^ | 19.2 | 0.0043 |
| miR-31-3p | 14.3 | 0.0044 |
| miR-31-5p | 16.0 | 0.005 |
| miR-486-5p | 16.2 | 0.041 |
| miR-187-3p | 10.2 | 0.083 |
| miR-196a | 0.221 | 0.097 |
| miR-126-3p | 8.3 | 0.11 |
| miR-503-5p | 5.13 | 0.33 |

^a^ microRNA highly expressed in blood, ^b^ pfp is for the rank product

test: percentage false positive predictions
