# Supplementary material for: Similar Squamous Cell Carcinoma Epithelium microRNA Expression in Never Smokers and Ever Smokers
Source: PLoS One. 2015 Nov 6;10(11):e0141695. doi: 10.1371/journal.pone.0141695 (PMC4636300; doi:10.1371/journal.pone.0141695)
Supplement: S4 Table — (DOC) [file pone.0141695.s004.doc]

**S4 Table** Ethanol Usage of Patients with OSCC in TCGA data set

| History of Ethanol Consumption | Ever Smoker | Never Smoker |
| --- | --- | --- |
| Yes | 158 (72.1%)^a^ | 46 (52.3%) |
| No | 58 (26.5%) | 41 (46.6%) |
| Not Available | 3 (1.4%) | 1 (1.1%) |

Ever smoker group shows more alcohol user with P< 0.001 based on Fisher Exact Test

|  | Ever Smoker | Never Smoker |
| --- | --- | --- |
| Average Alcohol Consumption Per Day | 3.64^a^ | 0.81 |

Ever smoker group shows higher alcohol consumption based on Student t-test t< 0.0
