# Supplementary material for: Similar Squamous Cell Carcinoma Epithelium microRNA Expression in Never Smokers and Ever Smokers
Source: PLoS One. 2015 Nov 6;10(11):e0141695. doi: 10.1371/journal.pone.0141695 (PMC4636300; doi:10.1371/journal.pone.0141695)

**S1 Fig. Comparison of miRNA expression profile from 1x and 9x dilution of a sample processed with Trizol and RNeasy chromatography shows no change.** Half of a single brush oral mucosal sample was diluted 9x in Trizol and then both halves were subjected to RT-PCR to quantify 13 different miRNAs. We show that for the methodology used, storage of the sample frozen in Trizol, followed by 1-bromo-3-chloropropane (BCP) phase separation, then immediate glass filter binding using RNeasy columns (Qiagen), the range of miRNA species recovered was uniform from a single sample. This occurred whether the same was concentrated or diluted 9x. MiRNA from the concentrated and diluted samples was converted to cDNA then quantified using RT-PCR. A comparison of Ct values for 13 detectable miRNA species revealed similar relative amounts of each species with a correlation coefficient 0.96.


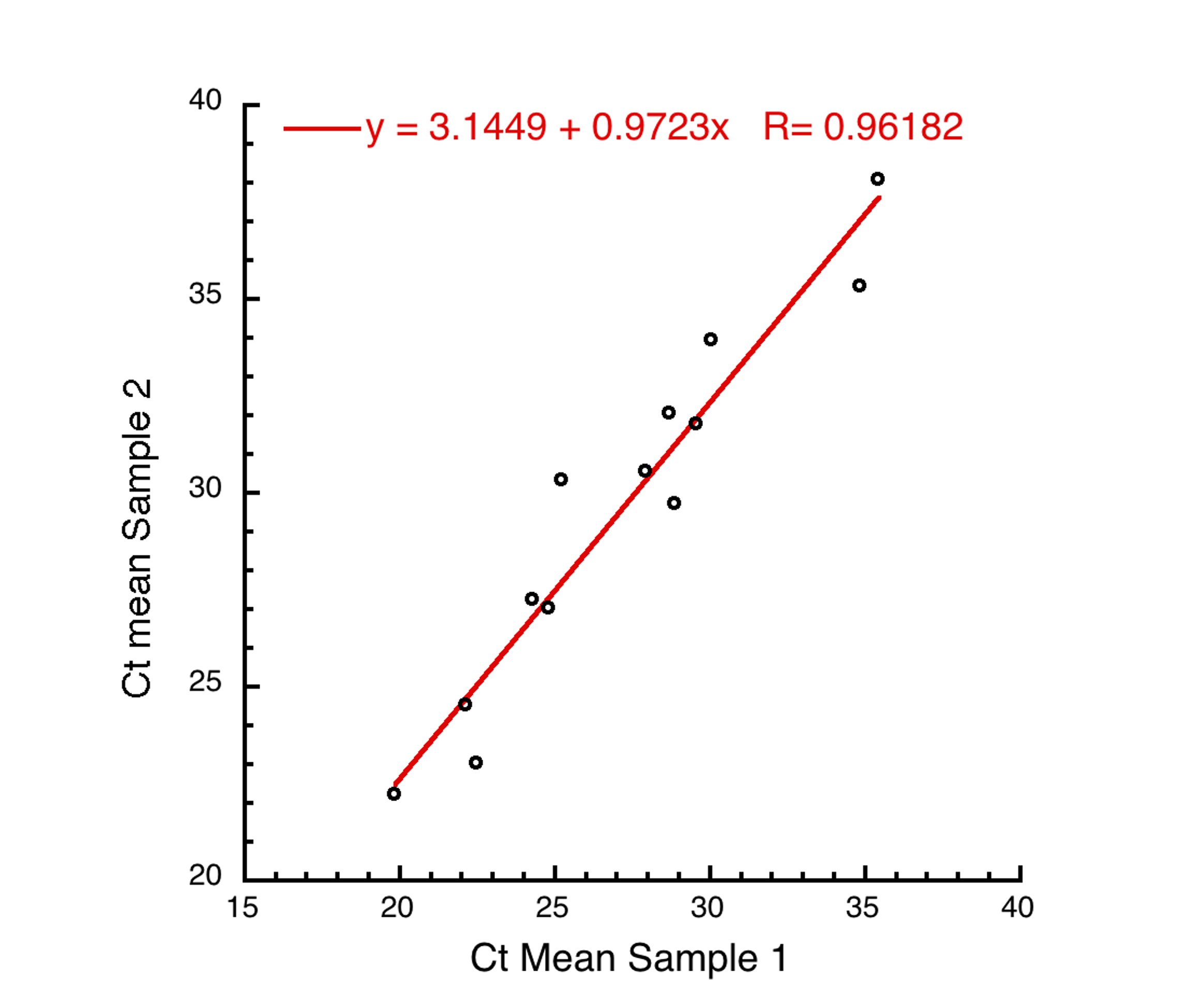

Supplement: S1 Fig — Half of a single brush oral mucosal sample was diluted 9x in Trizol and then both halves were subjected to RT-PCR to quantify 13 different miRNAs. We show that for the methodology used, storage of the sample frozen in Trizol, followed by 1-bromo-3-chloropropane (BCP) phase separation, then immediate glass filter binding using RNeasy columns (Qiagen), the range of miRNA species recovered was uniform from a single sample. This occurred whether the same was concentrated or diluted 9x. MiRNA from the concentrated and diluted samples was converted to cDNA then quantified using RT-PCR. A comparison of Ct values for 13 detectable miRNA species revealed similar relative amounts of each species with a correlation coefficient 0.96. (DOC) [file pone.0141695.s007.doc]
