# Supplementary figures and images for: Similar Squamous Cell Carcinoma Epithelium microRNA Expression in Never Smokers and Ever Smokers
Source: PLoS One. 2015 Nov 6;10(11):e0141695. doi: 10.1371/journal.pone.0141695 (PMC4636300; doi:10.1371/journal.pone.0141695)

Color Key

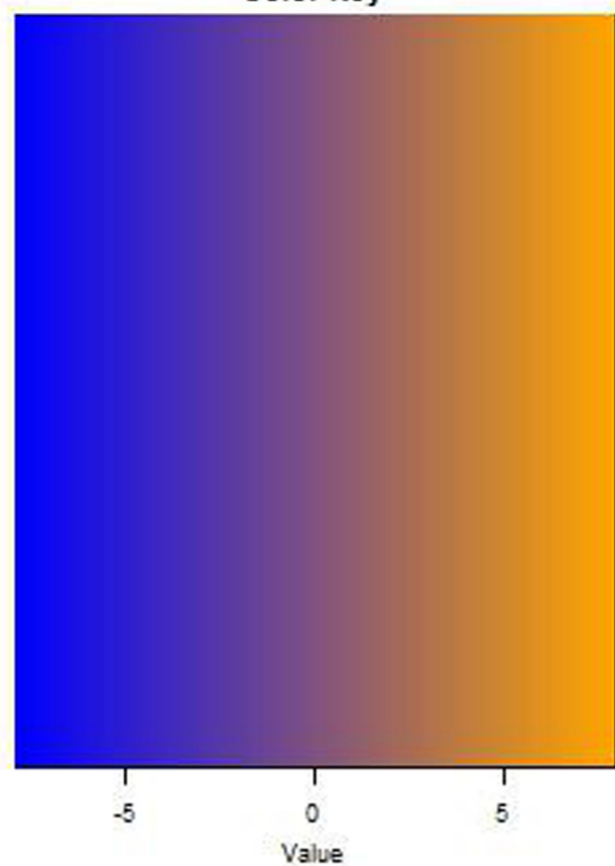

Heat map for NMF\_Gene Mean adjusted

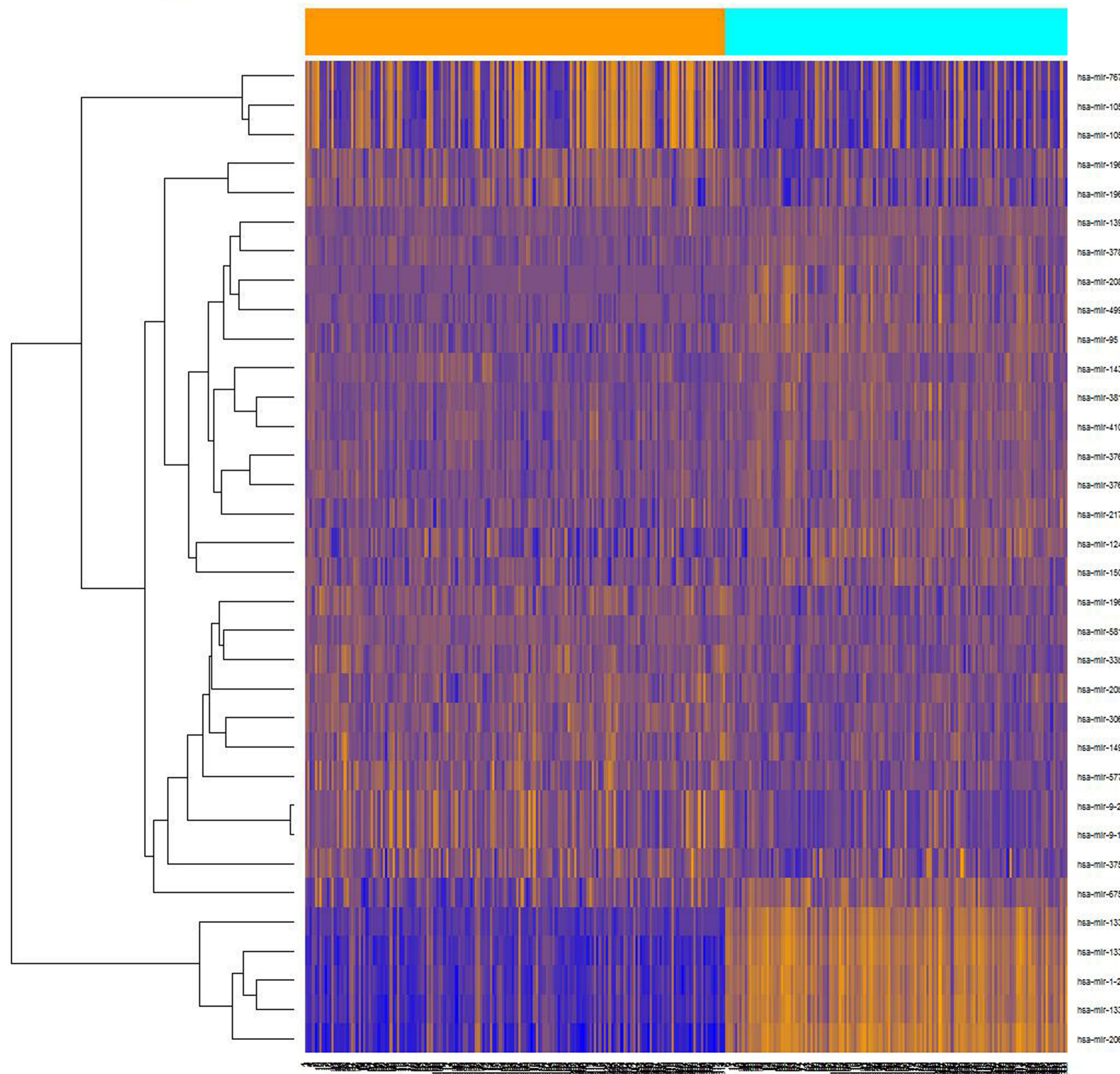

Supplement: S2 Fig — Sample clusters are identified by the colored horizontal bar. The color key provides information on relative expression levels. The relative expression levels of 50 variable miRNAs are shown. (PDF) [file pone.0141695.s008.pdf]
